# Supplementary material for: Clinical Characteristics and Outcomes of Extrapulmonary Nontuberculous Mycobacterial Infections in a Tertiary-Care Hospital: A Retrospective Study
Source: J Clin Med. 2024 Jul 26;13(15):4373. doi: 10.3390/jcm13154373 (PMC11313629; doi:10.3390/jcm13154373)
Supplement: Supplementary file 1 [file jcm-13-04373-s001.zip › jcm-3075312-supplementary.pdf]

Table S1. Clinical characteristics of 75 patients suspected of having NTM.

	Disease (N = 32)	Isolation (N = 43)	P [1] value
Fever	5 (16)	9 (21)	0.560
Duration of symptoms before evaluation, median (IQR), days	106.6 (30–135)	20 (3–60)	0.350
Symptoms			
Pain	21 (66)	27 (63)	0.800
Skin lesion (papules, nodule, patch, ulcer, mass)	7 (22)	1 (2)	0.009
Cellulitis-liked lesions	1 (3)	3 (7)	0.632
Graft stenosis	1(3)	0	0.427
Dyspnea	1 (3)	1 (2)	>0.999
General weakness	1 (3)	0	0.427
Nonspecific symptoms 2	0	4 (9)	>0.999
Symptoms associated with GI bleeding	0	3 (7)	0.256
Endoscopy or colonoscopy follow-up	0	2 (5)	0.504
Abdomen distension	0	2 (5)	0.504

Data are the number (%) of patients unless otherwise indicated. [1] Mann–Whitney U test or Fisher's exact test. P values < 0.05 were considered to be significant. [2] Nonspecific symptoms were defined as symptoms like hematuria, mental change, dizziness, and lymph adenopathy. These symptoms were grouped together, and these was one case for each symptom. Abbreviations: NTM, nontuberculous mycobacteria, IQR, interquartile range.

Table S2. Initial laboratory findings of patients with NTM disease.

Variable	Median (IQR)	Reference range
WBC (cells/μL)	7860 (6350–8280)	4000–11000
Neutrophil count (cells/μL)	4400 (3362.5–6192.5)	1700–7000
Lymphocyte count (cells/μL)	1445 (810–1797.5)	1000–4000
Hemoglobin (g/dL)	11.2 (10.2–12.7)	11.5–15.0
Platelet (103/μL)	309 (205–361)	140–400
CRP (mg/dL)	1.77 (0.38–4.9)	0–0.5

Abbreviations: NTM, nontuberculous mycobacteria; IQR, interquartile range; WBC, white blood cell; CRP, C-reactive protein.
